# COVID-19 underpinning the inverse equity hypothesis between public and private health care in Brazil

**DOI:** 10.62675/2965-2774.20240294-en

**Published:** 2024-03-25

**Authors:** Livia Maria Garcia Melro, Evelinda Marramon Trindade, Marcelo Park

**Affiliations:** 1 Hospital Samaritano Paulista Intensive Care Unit São Paulo SP Brazil Intensive Care Unit, Hospital Samaritano Paulista - São Paulo (SP), Brazil.; 2 Universidade de São Paulo Hospital das Clínicas Faculdade de Medicina São Paulo SP Brazil Health Technology Assessment Center, Hospital das Clínicas, Faculdade de Medicina, Universidade de São Paulo - São Paulo (SP), Brazil.; 3 Universidade de São Paulo Hospital das Clínicas Faculdade de Medicina São Paulo SP Brazil Intensive Care Unit, Hospital das Clínicas, Faculdade de Medicina, Universidade de São Paulo - São Paulo (SP), Brazil.

Since 1990, the Brazilian public health care system, known as the Unified Health System (SUS - *Sistema Único de Saúde*), has provided free health care services to all individuals throughout the country. However, approximately 24.9% of the Brazilian population has the financial means to afford private health care alternatives.^(1)^ Equity, a fundamental principle of SUS, has been extensively discussed in various dimensions of public health and ethics. Efforts have focused on investing in public health interventions to prevent further exacerbation of inequalities among less privileged individuals. For example, high-quality child public health programs have been widely accessible since 2000. Paradoxically, these programs are more frequently utilized by families in the Southern Region, who are in comparatively less need, as opposed to those in the Northeastern Region. This phenomenon has been referred to as the "inverse equity hypothesis".^(2)^ The introduction of new technologies may also have a considerable impact on exacerbating existing inequities.

The coronavirus disease 2019 (COVID-19) pandemic has placed an immense burden on the already limited structural, material, human resource, and financial capacity of SUS, leading to an increased in-hospital mortality rate.^(3)^ Nonetheless, despite numerous political and moral challenges, from a catastrophe management standpoint, the response of SUS has been remarkable, instilling trust and pride among its users.^(4)^ The pandemic has substantially increased the demand for intensive care unit (ICU) resources in Brazil, including the utilization of extracorporeal membrane oxygenation (ECMO) procedures. On May 13, 2021, the Brazilian Health Technology Assessment Committee (CONITEC - *Comissão Nacional de Incorporação de Tecnologias*) evaluated the potential inclusion of ECMO in SUS coverage during its fifth extraordinary meeting. However, the committee denied this coverage, citing the high cost and the potential exacerbation of inequities among different geographic regions in Brazil as the reasons for denial.^(5)^

**Figure 1:**
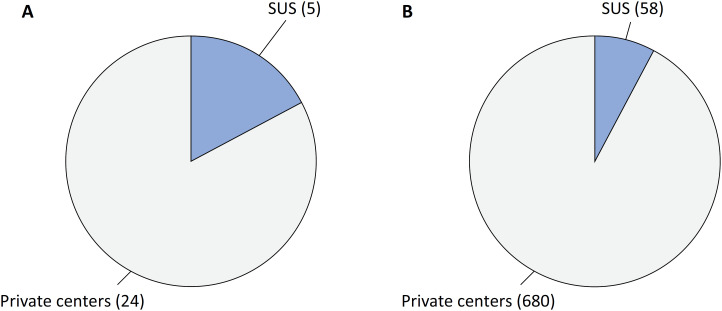
(A) Number of extracorporeal membrane oxygenation centers serving private or Unified Health System patients. (B) Number of patients who received extracorporeal membrane oxygenation support during the COVID-19 pandemic in Brazil, according to health care system funding.

In late 2021, an electronic survey was conducted involving 29 Brazilian adult ECMO centers, which yielded data on 738 individual ECMO procedures. The overall in-hospital mortality rate was 51%. Notably, this figure is likely an underestimation due to the limited sample size of hospitals, and there were also a few ECMO procedures performed in non-ECMO centers. Out of the 29 centers surveyed, only five (17%) catered to SUS patients. Interestingly, among the 738 ECMO procedures analyzed, only 58 (7.9%) were carried out for SUS patients (Figure 1), once again confirming the existence of the "inverse equity hypothesis" within the Brazilian health care system.^(5)^

SUS - Unified Health System (*Sistema Único de Saúde*); ECMO - extracorporeal membrane oxygenation.

In conclusion, ECMO is not a priority within the Brazilian public health system. However, it is crucial for Brazilian authorities and health care professionals to acknowledge the presence of the "inverse equity hypothesis," which was originally observed within the public health system but can also manifest between the public and private health care sectors. This inequity between the public and private systems undermines the principles of SUS and raises additional bioethical concerns, such as justice in Brazilian health care. Furthermore, this issue becomes even more important with the emergence of novel technologies in critical care, oncology, cardiology, rare diseases, and other medical fields.
