# Supplementary material for: COVID-19 underpinning the inverse equity hypothesis between public and private health care in Brazil
Source: Crit Care Sci. 2024 Mar 25;36:e20240294en. doi: 10.62675/2965-2774.20240294-en (PMC11098063; doi:10.62675/2965-2774.20240294-en)
Supplement: Translation

# Translation: Contribuição da COVID-19 à hipótese da equidade inversa entre a assistência médica pública e privada no Brasil

**DOI:** 10.62675/2965-2774.20240294-pt

**Authors:** Livia Maria Garcia Melro, Evelinda Marramon Trindade, Marcelo Park

**Affiliations:** 1 São Paulo SP Brasil Unidade de Terapia Intensiva, Hospital Samaritano Paulista - São Paulo (SP), Brasil.; 2 São Paulo SP Brasil Centro de Avaliação de Tecnologias em Saúde, Hospital das Clínicas, Faculdade de Medicina, Universidade de São Paulo - São Paulo (SP), Brasil.; 3 São Paulo SP Brasil Unidade de Terapia Intensiva, Hospital das Clínicas, Faculdade de Medicina, Universidade de São Paulo - São Paulo (SP), Brasil.

Desde 1990, o sistema público de saúde brasileiro, conhecido como Sistema Único de Saúde (SUS), oferece assistência médica gratuita a todos os indivíduos em todo o país. No entanto, aproximadamente 24,9% da população brasileira tem condições financeiras de pagar por alternativas de assistência médica privada.^(1)^ A equidade, um princípio fundamental do SUS, tem sido amplamente discutida em várias dimensões de saúde pública e ética. Os esforços têm se concentrado no investimento em intervenções de saúde pública para evitar o agravamento das desigualdades entre os indivíduos menos privilegiados. Por exemplo, programas de saúde pública infantil de alta qualidade têm sido amplamente acessíveis desde 2000. Paradoxalmente, esses programas são utilizados com mais frequência pelas famílias da Região Sul, que são comparativamente menos necessitadas, em comparação com as da Região Nordeste. Esse fenômeno tem sido chamado de “hipótese da equidade inversa”.^(2)^ A introdução de novas tecnologias também pode ter considerável impacto na exacerbação das desigualdades existentes.

A pandemia da doença pelo coronavírus 2019 (COVID-19) sobrecarregou imensamente a já limitada capacidade estrutural, material, de recursos humanos e financeira do SUS, levando a um aumento da taxa de mortalidade hospitalar.^(3)^ No entanto, apesar dos inúmeros desafios políticos e morais, do ponto de vista da gestão de catástrofes, a resposta do SUS tem sido notável, inspirando confiança e orgulho entre seus usuários.^(4)^ A pandemia aumentou substancialmente a demanda por recursos de unidade de terapia intensiva (UTI) no Brasil, incluindo a utilização de procedimentos de oxigenação por membrana extracorpórea (ECMO). Em 13 de maio de 2021, a Comissão Nacional de Incorporação de Tecnologias (CONITEC) avaliou a possível inclusão da ECMO na cobertura do SUS durante sua quinta reunião extraordinária. No entanto, o comitê negou essa cobertura, citando o alto custo e a potencial exacerbação das iniquidades entre as diferentes regiões geográficas do Brasil como os motivos da recusa.^(5)^

No fim de 2021, foi realizado um inquérito eletrônico com 29 centros brasileiros de ECMO para adultos, que produziu dados sobre 738 procedimentos individuais de ECMO. A taxa geral de mortalidade intra-hospitalar foi de 51%. Notavelmente, esse número é provavelmente uma subestimação, devido ao tamanho limitado da amostra de hospitais, alguns procedimentos de ECMO foram realizados em centros que não eram especializados em ECMO. Dos 29 centros pesquisados, apenas cinco (17%) atendiam a pacientes do SUS. Curiosamente, entre os 738 procedimentos de ECMO analisados, apenas 58 (7,9%) foram realizados em pacientes do SUS (Figura 1), confirmando, mais uma vez, a existência da “hipótese da equidade inversa” no sistema de saúde brasileiro.^(5)^

**Figura 1:** (A) Número de centros de oxigenação por membrana extracorpórea que prestam assistência a pacientes particulares ou do Sistema Único de Saúde. (B) Número de pacientes que receberam suporte de oxigenação por membrana extracorpórea durante a pandemia da COVID-19 no Brasil, conforme o financiamento do sistema de saúde.

SUS - Sistema Único de Saúde; ECMO - oxigenação por membrana extracorpórea.

Em conclusão, a ECMO não é uma prioridade no sistema de saúde pública brasileiro. No entanto, é fundamental que as autoridades brasileiras e os profissionais de saúde reconheçam a presença da “hipótese da equidade inversa”, que foi originalmente observada no sistema público de saúde, embora também possa se manifestar entre os setores público e privado de saúde. Essa iniquidade entre os sistemas público e privado prejudica os princípios do SUS e levanta outras preocupações bioéticas, como a justiça na assistência médica brasileira. Além disso, essa questão se torna ainda mais importante com o surgimento de novas tecnologias em terapia intensiva, oncologia, cardiologia, doenças raras e outras áreas médicas.
